# A novel variation in *DEPDC5* causing familial focal epilepsy with variable foci

**DOI:** 10.3389/fgene.2024.1414259

**Published:** 2024-06-21

**Authors:** Yanchi Wang, Wenbin Niu, Hao Shi, Xiao Bao, Yidong Liu, Manman Lu, Yingpu Sun

**Affiliations:** ^1^ Center for Reproductive Medicine, The First Affiliated Hospital of Zhengzhou University, Zhengzhou, China; ^2^ Henan Key Laboratory of Reproduction and Genetics, The First Affiliated Hospital of Zhengzhou University, Zhengzhou, China; ^3^ Henan Provincial Obstetrical and Gynecological Diseases (Reproductive Medicine) Clinical Research Center, The First Affiliated Hospital of Zhengzhou University, Zhengzhou, China; ^4^ Henan Engineering Laboratory of Preimplantation Genetic Diagnosis and Screening, The First Affiliated Hospital of Zhengzhou University, Zhengzhou, China

**Keywords:** disheveled, EGL-10, and pleckstrin domain-containing protein 5, focal epilepsy, gene variation, mini-gene splicing assay, mTORC1 pathway

## Abstract

**Background:**

Disheveled, EGL-10, and pleckstrin (DEP) domain-containing protein 5 (DEPDC5) is a component of GTPase-activating protein (GAP) activity toward the RAG complex 1 (GATOR1) protein, which is an inhibitor of the amino acid-sensing branch of the mammalian target of rapamycin complex 1 (mTORC1) pathway. GATOR1 complex variations were reported to correlate with familial focal epilepsy with variable foci (FFEVF). With the wide application of whole exome sequencing (WES), more and more variations in *DEPDC5* were uncovered in FFEVF families.

**Methods:**

A family with a proband diagnosed with familial focal epilepsy with variable foci (FFEVF) was involved in this study. Whole exome sequencing (WES) was performed in the proband, and Sanger sequencing was used to confirm the variation carrying status of the family members. Mini-gene splicing assay was performed to validate the effect on the alternative splicing of the variation.

**Results:**

A novel variant, c.1217 + 2T>A, in *DEPDC5* was identified by WES in the proband. This splicing variant that occurred at the 5′ end of intron 17 was confirmed by mini-gene splicing assays, which impacted alternative splicing and led to the inclusion of an intron fragment. The analysis of the transcribed mRNA sequence indicates that the translation of the protein is terminated prematurely, which is very likely to result in the loss of function of the protein and lead to the occurrence of FFEVF.

**Conclusion:**

The results suggest that c.1217 + 2T>A variations in *DEPDC5* might be the genetic etiology for FFEVF in this pedigree. This finding expands the genotype spectrum of FFEVF and provides new etiological information for FFEVF.

## Introduction

Focal epilepsy is a neurological condition in which the predominant symptom is recurring seizures that affect one hemisphere of the brain. It is the most commonly seen epilepsy, accounting for approximately 60% of all epilepsy cases (Dibbens et al., 2013; Zhang et al., 2021). It is increasingly recognized that focal epilepsy might have a genetic basis, and a number of mutations have been identified in autosomal dominant focal epilepsies in both ion-channel and non-ion-channel genes (Baulac, 2014; Steinlein, 2014). Among these genes, mutations in Disheveled, EGL-10, and pleckstrin (DEP) domain-containing protein 5 (*DEPDC5*) have been identified in 5% of sporadic epilepsy cases and 13% of familial epilepsy cases (Baulac, 2014; Tsai et al., 2017).

DEPDC5 plays a role in the GTPase-activating protein (GAP) activity toward RAG complex 1 (GATOR1), along with nitrogen permease regulator like-2 (NPRL2) and nitrogen permease regulator like-3 (NPRL3). The GATOR1 complex is a repressor of the mammalian target of rapamycin (mTOR) signaling pathway, which changes the nucleotide loading status (GTP or GDP) of the RAG proteins and deactivates them to release mTOR complex 1 (mTORC1) from the lysosome (Nicastro et al., 2017; Shen et al., 2018). The mTORC1 pathway is involved in regulating various important cellular processes, such as cell growth, protein synthesis, and autophagy, and its deregulation has been linked with diseases including cancer and epilepsy through the mutation of other repressors of the pathway (Meng et al., 2013; Lipton and Sahin, 2014; D’Gama et al., 2017). As an inhibitor of mTORC1, mutations in GATOR1 are predicted to cause epilepsy through mTORC1 hyperactivity, although the mechanism by which mTORC1 hyperactivity causes epilepsy and associated pathology needs further research (Baulac, 2016).

Here, we performed whole-exome sequencing (WES) for a male patient suffering from multiple unprovoked seizures and uncovered a novel variation (c.1217 + 2T>A) in the *DEPDC5* gene. This variation was inherited from his mother, who has a normal phenotype. The variation was verified to impact the alternative splicing of *DEPDC5* and introduce an early stop codon that resulted in the pre-maturation and loss of function of DEPDC5, which, as a consequence, might disturb the mTOR signaling pathway and lead to epilepsy. Our study expands the phenotype and genotype spectrum of DEPDC5 and further emphasizes the important role of *DEPDC5* in focal epilepsy.

## Materials and methods

### Patients

A family with a proband diagnosed with familial focal epilepsy with variable foci (FFEVF) was involved in this study. The family members taking part in this study were thoroughly informed about this study, and written informed consent was signed at their first consultation. This study was approved by the Ethics Committee of The First Affiliated Hospital of Zhengzhou University (Ethic no. 2019-KY-166).

### WES, variant interpretation, and Sanger sequencing

Genomic DNA (gDNA) was extracted from peripheral blood using the QIAamp DNA Blood Mini Kit (QIAGEN), following the manufacturer’s instructions. The gDNA was fragmented, and the sequencing library was prepared. Subsequently, target gene exons and DNA near the sheared region were captured and enriched using the BGI V4 chip, followed by mutation detection using the MGISEQ-2000 sequencing platform.

Mutations were annotated and screened based on patient’s clinical information, population databases, disease databases, and bioinformatic prediction tools. The classification of variation pathogenicity is based on the sequence variation interpretation guidelines of the American Society for Medical Genetics and Genomics (ACMG). “Ada” and “RF” scores were used to evaluate potential splicing variants predicted by dbscSNV (Jian et al., 2014). Variants identified through WES were confirmed by Sanger sequencing.

### Mini-gene construction

In order to construct the mini gene, we amplified the fragment of *DEPDC5* from exon 16 to the end of exon 18 by PCR and added the endonuclease recognition sequences of HindⅢ and NotⅠ to the front and end of the fragment, respectively. The PCR products were purified using alcohol and digested along with vector pCDNA3.1 (+) using endonucleases NotⅠ and HindⅢ (NEB). The digested PCR products and vector were purified via electrophoresis and ligated together with T4 ligase (NEB). Ligase products were transformed into DH5α competent cells, and transformed competent cells were plated on the LB plate coated with ampicillin. Then, single clones were picked up for proliferation and Sanger sequencing. Identified colonies were amplified, and plasmid DNA without endotoxin was extracted using the FastPure EndoFree Plasmid Maxi Kit (Vazyme). Primers used in this study are listed in Supplementary Material S1.

### Cell transfection

Human embryonic kidney 293T (HEK293T) cells were cultured in DMEM/high glucose (Gibco) using 10% FBS (Sigma) in a 37°C constant temperature water bath incubator at 5% CO_2_. The cells were dissociated into single cells using trypsin–EDTA (Thermo Fisher) after growth to an 80% confluence degree. Cells were counted, and 4 × 10^5^ cells were seeded in 6-well plates 24 h before transfection. The cell medium was changed using Opti-MEM (Thermo Fisher) 2 h before transfection. Lipofectamine 2000 (Thermo Fisher) was incubated for 20 min at room temperature and mixed thoroughly with 1 μg plasmid, and then the mixture was gently added to the cell medium. The cell medium was replaced by a fresh culture medium using 10% FBS 12 h after transfection.

### RT-PCR and Sanger sequencing

Total RNA was extracted 48 h after transfection using TRIzol reagent (Thermo Fisher) following the routine procedure. Retro-transcription was performed using HiScript III 1st strand cDNA Synthesis Kit with gDNA wiper (Vazyme) according to the manufacturer’s manual. cDNA was used as a template for PCR with primers pairs F3/R3 specifically binding to *DEPDC5* exons 16 and 18 (Supplementary Material S1). PCR products were analyzed via agarose gel electrophoresis and Sanger sequencing.

### Bio-informative analyses and 3D protein structure modeling

The *DEPDC5* gene sequences of different species were obtained from the NCBI Gene database (https://www.ncbi.nlm.nih.gov/gene/) and aligned by Molecular Evolutionary Genetics Analysis Version 11 (MEGA11) (Tamura et al., 2021).

The molecular structure of the cryo-EM structure of GATOR1 viewed by Mol* Viewer (Sehnal et al., 2021) was created by Shen et al. (2018) and stored at RCSB PDB (Berman et al., 2000). The structure of the mutated DEPDC5 protein was predicted and modeled using the SWISS-MODEL (Waterhouse et al., 2018) program.

## Results

### Case presentation

**FIGURE 1:**
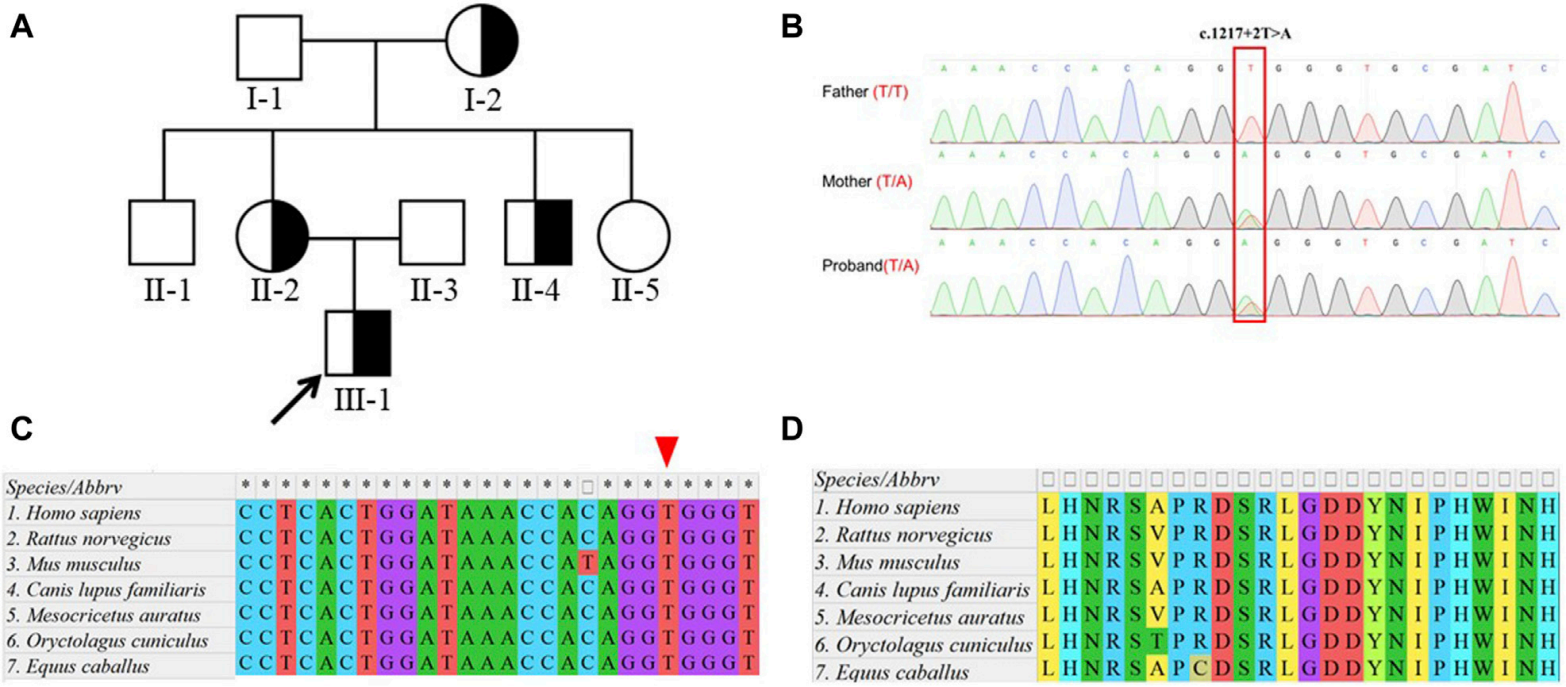
Identification of a heterozygous variation in *DEPDC5*. **(A)** Pedigree of the family. The proband (III-1) diagnosed with focal epilepsy is indicated by half-filled symbols with arrows. **(B)**. Sanger sequencing of the proband and his parents showed a c.1217 + 2T>A mutation (marked in the red box) in the *DEPDC5* gene, which was inherited from his mother. **(C)**. Species conservation analysis of DNA sequences around the variation site (marked by the red triangle) in *DEPDC5*. **(D)**. Species conservation analysis of amino sequences before the variation site (coded by exon 17) in DEPDC5.

The proband was a 7-year-old boy born following an uneventful full-term pregnancy. He presented with unprovoked seizures and dyskinesia and exhibited motor developmental delays such as the inability to walk or raise his head, along with high muscle tone and inconsistent limb activities on both sides. According to the mother’s account, the labor process went smoothly; however, antiepileptic drugs did not effectively control the symptoms during early childhood, resulting in a prolonged disease course. Screening for infection as well as blood and urine metabolic screening showed no abnormalities. The head MRI of the proband showed no obvious signal abnormalities. Both parents of the proband had normal phenotypes; one of the proband’s maternal uncles also had epilepsy, while one sister had normal phenotypes. Additionally, both maternal grandparents had normal phenotypes (Figure 1A).

### Identification of *DEPDC5* variation related to familial focal epilepsy with variable foci

To further elucidate the diagnosis and explore the etiology of the case, WES was performed, which identified a heterozygous variant (NM_001242897.1, exon 17, c.1217 + 2T>A) in the *DEPDC5* gene. The variant affected a canonical splicing site at the 5’ end of intron 17, potentially disrupting alternative splicing and leading to the loss-of-function effect on the protein. Sanger sequencing confirmed that this variant was inherited from his healthy mother, who did not exhibit any seizure-related symptoms (Figure 1B). The proband’s younger uncle (with epilepsy) and grandmother (without symptoms) also carried this variant (Supplementary Figure S1).

Notably, this variant was absent in the databases ESP6500 (the Genome Aggregation Database, V2), 1KGP (1000 Genomes Project, Phase3), ExAC (the Exome Aggregation Consortium, r0.3.1), and gnomAD (the Genome Aggregation Database, r2.0.1), indicating its rarity within these populations. The variant was also predicted to be splicing-influencing by “Ada” and “RF” scores (Table 1). Sequence alignments of different species showed that both the DNA loci and the amino acid residues upstream and downstream were evolutionarily conserved (Figures 1C, D). According to the ACMG guidelines, the novel variation c.1217 + 2T>A in our study was designated as likely pathogenic.

**TABLE 1:** Clinical examination and variant information.

Gene	Mutation	Inheritance	MAF				dbscSNV_ Ada_Score	dbscSNV_ RF_Score	Category
			ESP6500	ExAC	1,000 genomes	gnomAD			
*DEPDC5*	c.1217 + 2T>A	Inherited	NE	NE	NE	NE	0.9999	0.93	LP

Transcript, NM_001242897.1; MAF, minor allele frequency; NE, not exist; LP, likely pathogenic.

### Functional splicing examination of the variant with mini-gene splicing assays

**FIGURE 2:**
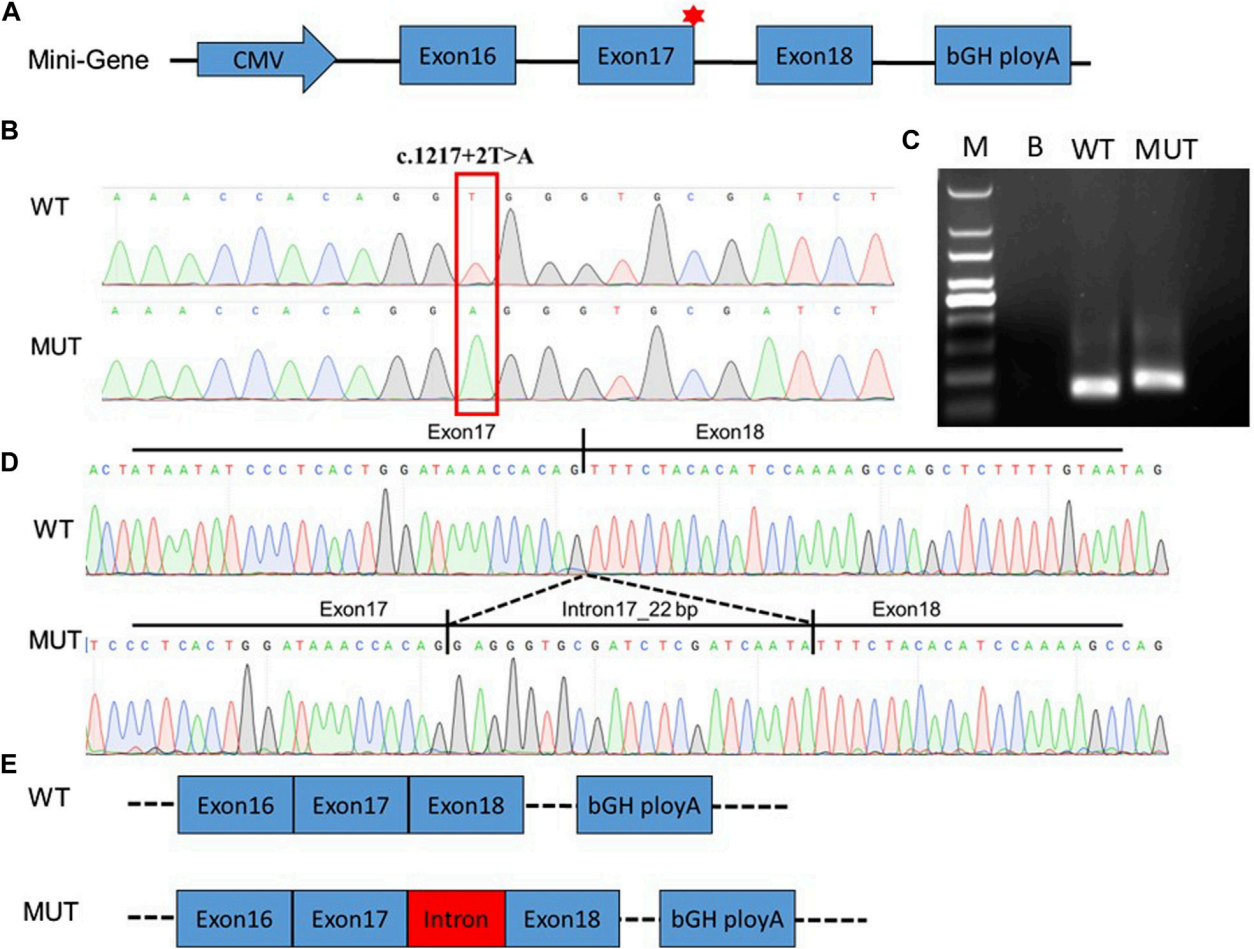
Functional splicing examination of the variant with mini-gene splicing assays. **(A)**. Schematic diagram of the constructed mini-gene. **(B)**. Sanger sequencing confirmed that wild-type and mutant fragments were successfully introduced into the mini-gene construct. The splicing variation c.1217 + 2T>A in *DEPDC5* is indicated by the red box. **(C)**. RT-PCR was performed to verify alternative splicing in the wild-type and mutant groups. Abnormal splicing bands in mutant groups were uncovered in HEK 293T cells. **(D)**. Alternative splicing was affected by the c.1217 + 2T>A variation in *DEPDC5*. PCR product sequencing revealed that 22 bp of intron 17 retained without exon skipping. **(E)**. Alternative schematic diagram. WT, wild type; MUT, mutant type; M, 100-bp DNA ladder; B, blank.

To validate the effect of the c.1217 + 2T>A variation on RNA alternative splicing, we conducted a mine-gene splicing assay by constructing expression vectors containing the wild-type (WT) and mutation-type (MUT) target DNA fragments (Figure 2A). The vectors were confirmed by Sanger sequencing (Figure 2B), and plasmid DNA without endotoxin was transfected into HEK 293T cells. The total RNA of transfected cells was extracted, and reverse transcription was performed to achieve the cDNA. Agarose electrophoresis of RT-PCR products revealed two distinct splicing patterns (Figure 2C), and Sanger sequencing uncovered abnormal splicing that occurred in cells transfected with the mutation plasmid. Sequencing analysis showed that a 22-bp intron 17 fragment was retained in the mutation group (Figures 2D, E).

### Translation analysis and protein modeling

**FIGURE 3:**
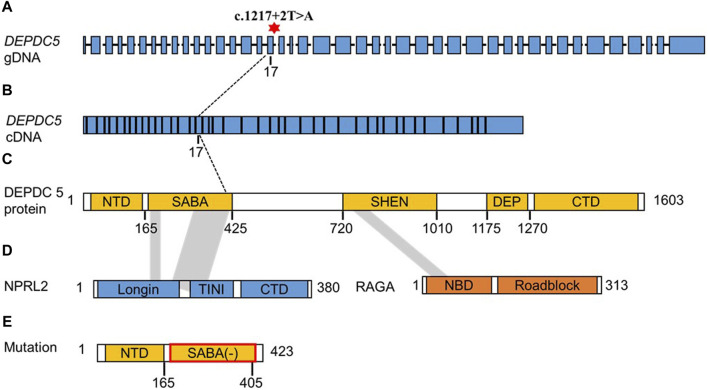
Schematic diagram structure of the *DEPDC5* gene, protein domain, and interaction with NPRL2 and RAGA. **(A)**. gDNA structure of DEPDC5. The red asterisk represents the variation site c.1217 + 2. **(B)**. cDNA structure of DEPDC5. **(C)**. Schematic diagram of the DEPDC5 domain. DEPDC5 protein contains the NTD, SABA domain, SHEN domain, DEP domain, and CTD. The shadow showed the region interacted with NPRL2 and RAGA. **(D)**. Schematic diagram of NPRL2 and RAGA structures. The NPRL2 protein contains an N-terminal longin domain (Longin), a tiny intermediary of NPRL2 that interacts with the DEPDC5 domain (TINI) and a CTD. **(E)**. Schematic diagram of the predicted protein structure of mutated DEPDC5.

**FIGURE 4:**
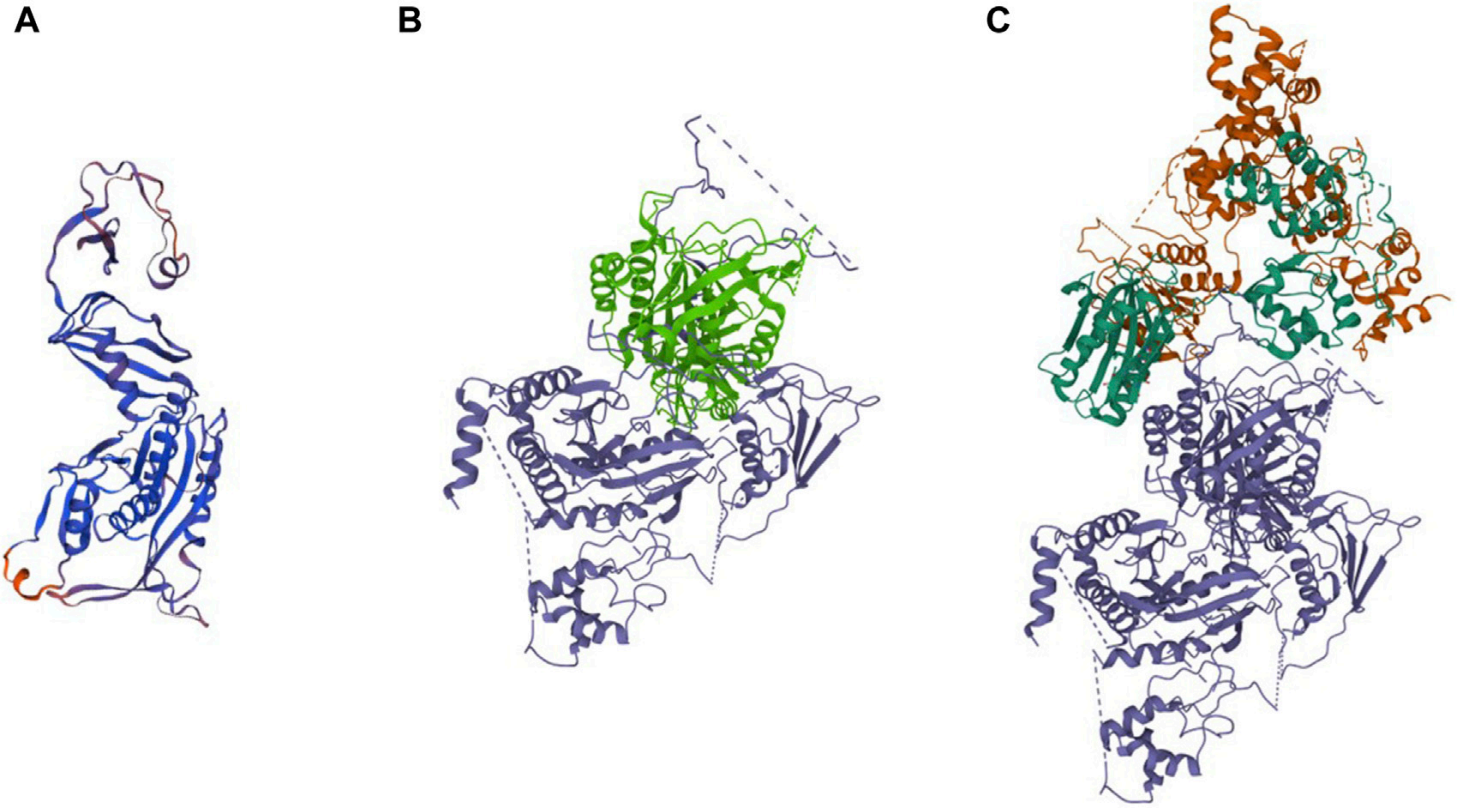
Predicted 3D protein structure of the DEPDC5 variant and the cryo-EM structure of WT DEPDC5 and GATOR1 complex. **(A)**. Predicted variant protein structure. **(B)**. Cryo-EM structure of the WT protein. The light green part represents the remaining part of the variant DEPDC5. **(C)**. Cryo-EM structure of the GATOR1 complex. The blue parts represent DEPDC5, the green parts represent NPRL2, and the brown parts represent NPRL3.

We further analyzed the cDNA sequence of *DEPDC5* with variation and found that the retention of the 22-bp fragment led to frameshift variation and the early appearance of the stop codon, which resulted in the pre-maturation of DEPDC5 with 423 amino acid residues. Thus, the variation could be described as c.1217 + 2T>A (p.S406Rfs*18). Mutated DEPDC5 lost the whole enhancement of nucleotidase activity (SHEN) domain, Disheveled, EGL-10, and pleckstrin (DEP) domain, and C-terminal domain (CTD), and the end of the structural axis for binding arrangement (SABA) domain also changed, as shown by the SABA (−) labeled in the red frame in Figure 3. According to the previous study, the SHEN domain contacts the nucleotide-binding domain of RAGA, and the CTD is located in the core of DEPDC5 and contacts all the other domains of DEPDC5 except the N-terminal domain (NTD) (Figure 3), making it the central organizer of this multi-domain protein (Shen et al., 2018). Loss of these domains means a radical change in the protein, and its function would be severely compromised. We could intuitively observe the significant alteration of DEPDC5 brought by the gene variation through the 3D model comparison of the mutated protein with the wild type (Figure 4).

## Discussion

In this article, we described a patient with a clinical presentation compatible with the *DEPDC5* phenotype, and we discovered a germline splicing variant c.1217 + 2T>A in the *DEPDC5* gene. As far as we know, this splicing variant has not been recorded or reported before. Approximately 9% of all mutations reported in the Human Gene Mutation Database (HGMD) are splicing mutations (18761/208368), and this number may be underestimated (Anna and Monika, 2018). Accurate pre-mRNA splicing is critical for proper protein translation, and it depends on the existence of consistent cis sequences that define exon–intron boundaries and regulatory sequences recognized by splicing mechanisms. Point mutations in these consistent sequences may result in the improper recognition of exons and introns and may lead to the formation of abnormal transcripts of mutant genes. However, the exact effect of specific splicing mutations on alternative pre-mRNA splicing needs further validation. The alternative splicing that does not result in the frameshift might have a mild effect on protein function. Mutations in the canonical splice sequences usually lead to single-exon skipping; however, in our study, unlike the skipping of exon 17 as expected, a segment of intron 17 was included in mRNA due to the variation. One possible reason for this may be that the splicing site is weak, while the presence of mutations uncovers the cryptic splice site in the neighboring intron, and this alternative site is used in the splicing process (Anna and Monika, 2018). The retention of the intron sequence resulted in a frameshift and the early appearance of a stop codon, causing the *DEPDC5* protein to be pre-maturated and lose its normal function.

*DEPDC5* (OMIM 614191) is located on chromosome 22q12.3 and is 154 kb in length. The DEPDC5 protein contains 1,603 amino acids and can be divided into five domains, which were named—in order from the N terminus to the C terminus—the NTD, (SABA) domain, steric hindrance for the SHEN domain, DEP domain, and CTD (Figure 3C). (Shen et al., 2018) DEPDC5 interacts with the NPRL2–NPRL3 heterodimer through the SABA domain and forms the GATOR1 complex, while the GATOR1 complex interacts with RAGA through the SHEN domain of DEPDC5. The variation in this study led to an alternative splice and might produce a truncated protein with 423 amino acids. Even if the truncated protein could be stable *in vivo*, the protein lost the SHEN and CTD domains; so, the GATOR1 complex formed with the truncated protein might lose its GAP activity. Thus, this splicing variant was highly likely to result in haploinsufficiency.

Haploinsufficiency is the likely mechanism underlying the pathogenesis of *DEPDC5* mutations, and epilepsy related to *DEPDC5* variants is inherited in an autosomal dominant manner. Nevertheless, asymptomatic carriers are commonly seen in *DEPDC5*-related families, and the incomplete penetrance differs among different pedigrees (Baldassari et al., 2019; Liu et al., 2020). Incomplete penetrance is probably due to the combination of genetic, environmental, and lifestyle factors (Coll et al., 2018). Furthermore, this might also result in the additive effect of multiple independent variations, which can interfere with and often increase the severity of the phenotype. Ingrid et al. first proposed the second-hit hypothesis in *DEPDC5* mutation-related epilepsy (Scheffer et al., 2014). According to the hypothesis, in addition to the germline mutations, there is also a somatic mutation on the other allele or on another gene involved in the mTOR pathway in local brain tissue (Ribierre et al., 2018; Lee et al., 2019). Though there are already a few cases supporting this hypothesis, further exploration is still needed.

Nevertheless, there are also some limitations to this study. For instance, some clinical materials were already not suitable for special tests, like RNA analysis or Western blotting, when we started the study, as this was a retrospective study. So, the results of the mini-gene splicing assay were not further validated in patients’ samples.

In conclusion, in this article, we describe a pedigree with FFEVF harboring a novel splicing variation, c. 1217+2T>A (p.S406Rfs*18), in the *DEPDC5* gene. The effect of this variation on alternative splicing and translation was confirmed by mini-gene splice assays and the following analysis. Our study provided a new source of evidence for the pathogenicity of splicing variation in the GATOR1 complex and expanded the phenotype and genotype spectrum of FFEVF, enhancing the critical role of *DEPDC5* in neurodevelopment.

## Data Availability

The datasets presented in this study can be found in online repositories (https://figshare.com/articles/dataset/DataSheet1_xlsx/26021818). The names of the repository/repositories and accession number(s) can be found in the article/Supplementary Material.
