# Supplementary figures and images for: A novel variation in DEPDC5 causing familial focal epilepsy with variable foci
Source: Front Genet. 2024 Jun 21;15:1414259. doi: 10.3389/fgene.2024.1414259 (PMC11227254; doi:10.3389/fgene.2024.1414259)

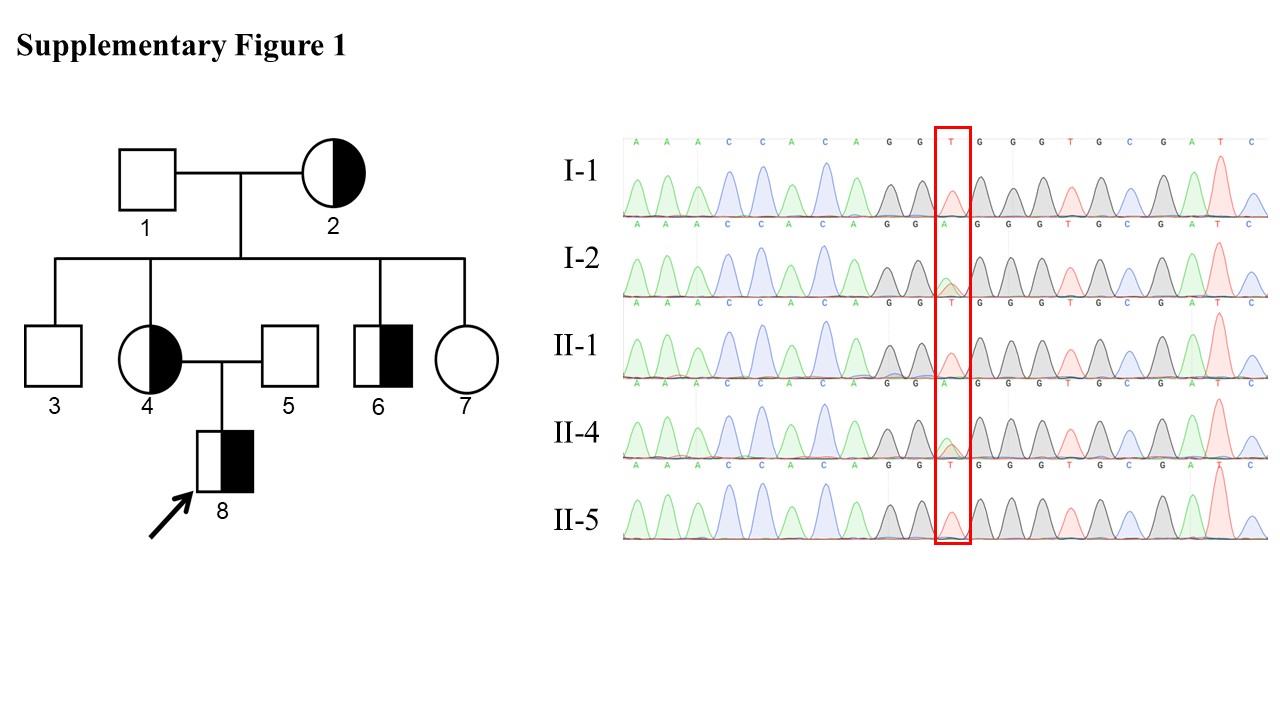

Supplement: Supplementary file 1 [file Image1.JPEG]
