# Supplementary material for: A novel variation in DEPDC5 causing familial focal epilepsy with variable foci
Source: Front Genet. 2024 Jun 21;15:1414259. doi: 10.3389/fgene.2024.1414259 (PMC11227254; doi:10.3389/fgene.2024.1414259)
Supplement: Supplementary file 2 [file DataSheet1.PDF]

Primers sequences

Primers for identification of mutation sites:

F1	5’	GGCTGGTCTTGAACACCTGATT	3’
R1	5’	GGGAGTTGGGAATAGAAGGACAT	3’

Primers for amplifying the fragment of *DEPDC5* from exon 16 to the end of exon 18 by PCR, and added the endonuclease recognition sequences of HindIII and NotI to the front and end of the fragment, respectively:

F2	5’	CCCAAGCTTGAATTGGTGTGGATTTGGTGT	3’
R2	5’	TAAACTATCGGCCGCCTTCTTTCCTGCCAGTTTTA	3’

Primers for amplification of cDNA products:

F3	5’	ACCGTTACATGCTGTCCCATT	3’
R3	5’	TTTCCTGCCAGTTTTATTCGTG	3’
